# Supplementary material for: Changes in Phenolic Compounds and Antioxidant Activity during Development of ‘Qiangcuili’ and ‘Cuihongli’ Fruit
Source: Foods. 2022 Oct 13;11(20):3198. doi: 10.3390/foods11203198 (PMC9602302; doi:10.3390/foods11203198)
Supplement: Supplementary file 1 [file foods-11-03198-s001.zip › foods-1969115-supplementary.pdf]

Table S1. Mobile phase ratio and elution description.

Time/min	A%	B%
0	95	5
50	72	28
60	57	43
60-65	57	43
70-75	95	5

Table S2. Primer sequences for real-time PCR.

Gene Name	Forward Primer Sequence (5′-3′)	Reverse Primer Sequence (5′-3′)	Product Length (bp)
18s	GTTACTTTTAGGACTCCGCCA	ATTCCTTTAAGTTTCAGCCTTG	97
PAL1	GATGAATCTATCTGCTCCTT	GTTCTTGCTTCTGTCTCA	105
PAL3	CAGTGCTACCTATCCATT	AGATTGAAGTGCTTGAAC	98
C4H	TCTTGAGAGGCTACTTGA	GCACTTCAGTCCTTCATT	131
4CL1	CTGCTGTTGTACCTATGA	TATTGCTTGATGTCCTCTT	103
4CL2	TGCTGTCAATGTGCTTAG	TCCACGATCTTCATCTCA	105
CHS	GCCACAAGACACATACTA	CTTCCTAACCTCATCCAA	78
HCT1	GAACATCGTGTAGCAGAT	AATGGTGGAATTGTAAGGT	92
HCT2	AATACCAACCTTCTCCACAA	GACCTGCCAACATCTCAA	180
HCT3	GGCTGGCTTTCGTAATAC	ATATGTCGTATAACAACTTGGAA	108
